# The hippocampus of the eastern rock sengi: cytoarchitecture, markers of neuronal function, principal cell numbers, and adult neurogenesis

**DOI:** 10.3389/fnana.2013.00034

**Published:** 2013-10-29

**Authors:** Lutz Slomianka, Tanja Drenth, Nicole Cavegn, Dominik Menges, Stanley E. Lazic, Mashudu Phalanndwa, Christian T. Chimimba, Irmgard Amrein

**Affiliations:** ^1^Institute of Anatomy, University of ZürichZürich, Switzerland; ^2^In Silico Lead Discovery, Novartis Institutes for Biomedical ResearchBasel, Switzerland; ^3^Mammal Research Institute, Department of Zoology and Entomology, University of PretoriaHatfield, South Africa; ^4^Western Cape Nature Conservation Board (CapeNature)Cape Town, South Africa; ^5^Department of Science and Technology-National Research Foundation Centre of Excellence for Invasion Biology, Department of Zoology and Entomology University of PretoriaHatfield, South Africa

**Keywords:** Macroscelididae, comparative neuroanatomy, calcium-binding proteins, somatostatin, correspondence analysis, dentate gyrus, proliferation, neuronal differentiation

## Abstract

The brains of sengis (elephant shrews, order *Macroscelidae*) have long been known to contain a hippocampus that in terms of allometric progression indices is larger than that of most primates and equal in size to that of humans. In this report, we provide descriptions of hippocampal cytoarchitecture in the eastern rock sengi (*Elephantulus myurus*), of the distributions of hippocampal calretinin, calbindin, parvalbumin, and somatostatin, of principal neuron numbers, and of cell numbers related to proliferation and neuronal differentiation in adult hippocampal neurogenesis. Sengi hippocampal cytoarchitecture is an amalgamation of characters that are found in CA1 of, e.g., guinea pig and rabbits and in CA3 and dentate gyrus of primates. Correspondence analysis of total cell numbers and quantitative relations between principal cell populations relate this sengi to macaque monkeys and domestic pigs, and distinguish the sengi from distinct patterns of relations found in humans, dogs, and murine rodents. Calretinin and calbindin are present in some cell populations that also express these proteins in other species, e.g., interneurons at the stratum oriens/alveus border or temporal hilar mossy cells, but neurons expressing these markers are often scarce or absent in other layers. The distributions of parvalbumin and somatostatin resemble those in other species. Normalized numbers of PCNA+ proliferating cells and doublecortin-positive (DCX+) differentiating cells of neuronal lineage fall within the overall ranges of murid rodents, but differed from three murid species captured in the same habitat in that fewer DCX+ cells relative to PCNA+ were observed. The large and well-differentiated sengi hippocampus is not accompanied by correspondingly sized cortical and subcortical limbic areas that are the main hippocampal sources of afferents and targets of efferents. This points to intrinsic hippocampal information processing as the selective advantage of the large sengi hippocampus.

## INTRODUCTION

The small family of sengis or elephant shrews (Macroscelididae) was long placed within the order Insectivora, but is now considered to form an order by itself, the Macroscelidea (Rathbun, 2009). They have been positioned with the Anagalida (rodents and lagomorphs) or even been taken as living representatives of Condylarths, the predecessors of hoofed mammals (Holroyd and Mussell, 2005). Molecular studies point to other mammals now endemic to Africa (Afrotheria/Tethytheria, e.g., elephants, sirenians, aardvarks, and hyraxes) as their closest relatives (Smit et al., 2011). Sengis are generally small sized mammals and, with the exception of one species, they inhabit woods, bushland and dry boulder or gravel plains of the southern part of the continent (Rathbun, 2005, 2009). The radiation that gave rise to extant species dates to only ~11 million years ago (Smit et al., 2011), i.e., at about the time when also the house mouse and rat may have diverged (Michaux et al., 2002). Sengis live in facultative monogamy, a rare trait in mammals, and give birth to highly precocial young.

Based on a phylogenetic analysis of gross characters of the brain, the rufous sengi (*Elephantulus rufescens*) associated with insectivores (Johnson et al., 1982; Kirsch and Johnson, 1983). However, Stephan and colleagues (Stephan and Spatz, 1962; Stephan and Andy, 1964) noted the prominent development of olfactory and subcortical visual centers. Based on these and other morphological observations and reflecting the later taxonomic changes, Stephan grouped the dusky footed sengi (*Elephantulus fuscipes*) and checkered sengi (*Rhynchocyon cirnei*) as macrooptic insectivores, which clearly deviated from his basal and higher insectivores in the relative size of the entire brain as well as that of major brain divisions. The most notable deviation was found for the hippocampus, which is 3.5 times larger than that of basal insectivores (Stephan, 1961; Stephan and Andy, 1964), larger than that of most primates, and equal in relative size to that of humans (Stephan, 1983). This observation confirmed the rather graphic early description of the appearance of sengi brains, including the eastern rock sengi (*Elephantulus myurus*) that we investigated, by Le Gros Clark (1928), who found the hippocampus to be an “enormous mass,” “hypertrophied,” and “immense and seemingly bizarre” developed. The few later studies of sengi neuroanatomy can still be comprehensively reviewed. Sanides and Sanides (1974) grouped *Elephantulus* with insectivores based on the large size of neocortical stellate cells and their extensive dendritic arbors when compared to rodents and primates. Sensory cortices of the cape sengi (*Elephantulus edwardii*), with emphasis on the somatosensory cortex, were mapped, found to be well-developed, yet without the cytochrome oxidase signature that allows the anatomical identification of these areas in other mammals (Dengler-Crish et al., 2006). Based on the distributions of cortical interneuron types characterized by the presence of calcium-binding proteins, of non-phosphorylated neurofilament containing neurons and glial fibrillary acidic protein (GFAP) expression, sengis, represented by a single specimen of the black and rufous sengi (*Rhynchocyon petersi*), were grouped with other afrotherian and xenarthran species and suggested to express distributions observed early in mammalian cortical evolution (Sherwood et al., 2009). A survey of the eastern rock sengi serotonergic, catecholaminergic, and cholinergic systems revealed an unusual presence of presumptive cholinergic neurons in the superior and inferior colliculi and cochlear nucleus, while other aspects of the distribution of markers for these systems did not deviate grossly from patterns observed in other eutherian mammals (Pieters et al., 2010). Notably, there have been no studies that followed up on what makes the sengi forebrain stand out among other mammals – their very large hippocampus.

In this study, we provide a general description and discussion of the cytoarchitecture of the hippocampus of the eastern rock sengi, the numbers of its principal neurons and the distribution of interneuron populations characterized by the expression of calcium-binding proteins and somatostatin. Of particular interest to us is how a very large hippocampus impacts on adult hippocampal neurogenesis (AHN). The observations that, in Murinae, a large habitat can be associated with either a large dentate gyrus and a low level of AHN or with a smaller dentate gyrus and a high level of AHN (Amrein et al., 2004a,b) suggest that species of this group may pursue different strategies to satisfy dentate information processing needs. Low levels of AHN in primates (Eriksson et al., 1998; Kornack and Rakic, 1999; Jabès et al., 2010; Amrein et al., 2011; Marlatt et al., 2011) may find their explanation in their large hippocampus (Stephan, 1975, 1983). This idea was put to the test in the sengi dentate gyrus by assessing cell proliferation, neuronal differentiation, and cell death.

## MATERIALS AND METHODS

### ANIMALS

**FIGURE 1:**
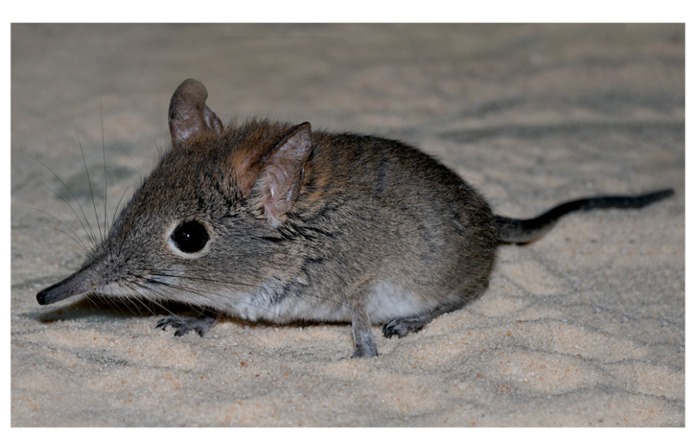
**The eastern rock sengi, *Elephantulus myurus* (courtesy of Heike Lautermann, Department of Zoology, University of Pretoria, South Africa)**.

Seven female (body weight: 44–62 g) and seven male (body weight: 40–56 g) eastern rock sengis (*Elephantulus myurus*, **Figure 1**) were caught in Sherman life traps at the Goro Game Reserve, Limpopo Province, South Africa (Permit 0089-CPM-401-00004, CITES and Permit Management Office, Department of Environmental Affairs, Limpopo Province). Tissues were harvested from animals euthanized under projects in accord with the ethics guidelines of South Africa (University of Pretoria Clearance EC028-07) and the guidelines of the American Society of Mammalogists (Gannon et al., 2007). After trapping, animals were deeply anesthetized with pentobarbital (50 mg/kg), weighed and perfused transcardially using heparinized, cold phosphate buffer saline (PBS, pH 7.4) followed by 0.6% sodium sulfide in phosphate buffer and, finally, cold 4% paraformaldehyde solution in PBS with 15% picric acid (PFA-PA). The brains were removed, weighed, separated into hemispheres and post-fixed overnight. Thereafter all right hemispheres and two left hemispheres were transferred to 30% sucrose solution for cryoprotection. The remaining left hemispheres were conserved in fresh PFA-PA for plastic embedding.

### HISTOLOGY AND IMMUNOHISTOCHEMISTRY

The left hemispheres of 12 animals were dehydrated and embedded in glycol methacrylate (Technovit 7100, Heraeus Kulzer GmbH, Wehrheim/Ts, Germany) according to the manufacturer’s instructions but with extended infiltration times (Amrein and Slomianka, 2010). Series of every sixth 20 μm thick horizontal section were mounted, one series was Giemsa-stained (Giemsa stock solution 1.09204.0500, Merck, Darmstadt, Germany) following the protocol of Iñiguez et al. (1985) and one series of two animals was Timm-stained (Danscher and Zimmer, 1978).

Twelve series of 40 μm thick frozen sections were cut from the right hemispheres (12 sagittal, one coronal and one horizontal) and two left hemispheres (one coronal and one horizontal). Sections were collected in cryoprotectant and stored at -20°C until further processing. Details of the immunohistochemical procedures (antibody, source, dilution, antigen, antigen retrieval) are listed in **Table 1**. Between all steps, sections were washed with Tris-Triton (TBS pH 7.4 with 0.05% Triton) and, after incubation with primary antibody, with TBS only. After preincubation with 2% normal serum, 0.2% Triton, and 0.1% bovine serum albumin in TBS, sections were incubated with the primary antibody overnight at 4°C. Incubation in secondary antibody (1:300) and ABC solution (Vectastain Elite Kits, Vector Laboratories, Burlingame, CA, USA) followed the manufacturer’s instructions. Finally, sections were diaminobenzidine-stained, dehydrated and mounted.

**Table 1:** Antigen specific details of the immunohistochemical procedures.

Antibody	Source	Antigen	Dilution	Antigen retrieval	Peroxidase block
Polyclonal rabbit anti-**calbindin** IgG	CB-38a, Swant	Recombinant rat calbindin	1:10000	See calretinin	See calretinin
Monoclonal mouse anti-**calretinin** IgG	MAB1568, Milipore	Recombinant rat calretinin	1:500	3 × 15 s microwaved in citrate buffer pH6.0 (Target Retrieval Solution, DAKO)	15 min in 0.6% H_2_O_2_ in TBS-Triton
Polyclonal goat anti-**doublecortin** IgG	sc-8066, Santa Cruz Biotechnology	Epitope mapping at the C-terminus of human doublecortin	1:5000	See calretinin	See calretinin
Polyclonal goat anti-**NeuroD** IgG	sc-1084, Santa Cruz Biotechnology	Epitope mapping to the N-terminus of mouse NeuroD	1:1000	See PCNA	None
Polyclonal rabbit anti-**PCNA** IgG	sc-7907, Santa Cruz Biotechnology	Synthetic full-length human PCNA	1:200	40 min at 95°C in citrate buffer pH6.0 (Target Retrieval Solution, DAKO)	None
Monoclonal mouse anti-**parvalbumin** IgG	P-3171, Sigma	Carp muscle parvalbumin	1:20000	None	See calretinin
Monoclonal mouse anti-**PSA-NCAM** IgG	MAB5324, Millipore	Viable Meningococcus group B (strain 355)	1:10000	None	See calretinin
Polyclonal rabbit anti-**somatostatin** IgG	ab103790, abcam	Synthetic peptide corresponding to amino acids 1–14 of human somatostatin	1:500	See calretinin	See calretinin

For all antigens, mouse forebrain sections were processed in the same batch. None of the antibodies stained structures that, based on concurrently processed mouse sections and published data on the distributions of the antigens, were regarded unspecific. Several attempts to stain proliferating cells using the marker Ki67 failed. A similar failure was noted for another Afrotherian species (Patzke et al., 2013).

### QUANTITATIVE PROCEDURES

**FIGURE 2:**
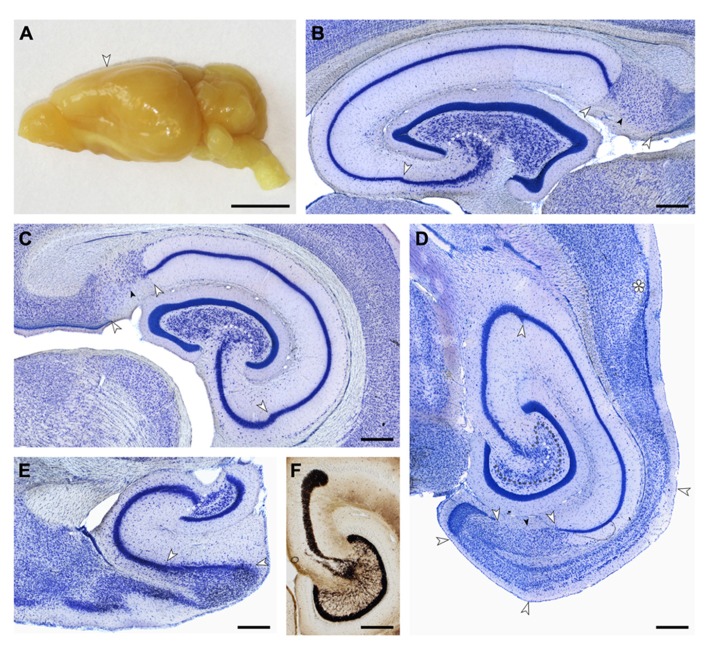
**(A)** Macroscopic lateral view of the eastern rock sengi brain. The posterior expansion of the hemisphere largely reflects the shape of the underlying hippocampus. Scale bar: 5 mm. **(B–E)** Light arrows and light dotted line mark the borders between hippocampal fields. Small dark arrows mark the border between the proximal and distal parts of the subiculum. **(B,C,E)** 40 μm-thick frozen sections; **(D,F)** 20 μm-thick plastic embedded section. Scale bars in **(B–F)** 0.5 mm. **(B)** Sagittal section of the septal hippocampus located at the transition from the first to the second hippocampal quarter. **(C)** Frontal section of the hippocampus located midway along the septotemporal hippocampal axis. **(D)** Horizontal section of the temporal hippocampus located at the transition from the third to the fourth hippocampal quarter. The dark dotted line marks the tentative border between the reflected blade of CA3 and the mossy cell layer. Asterisk: sectioning artifact. **(E)** Sagittal section of the temporal pole of the hippocampus. The plane of section corresponds to **(B)**. **(F)** Timm-stained section corresponding to **(D)** showing a dense, narrow band of staining below the granule cell layer from which individual strands extend to coalesce into the CA3 mossy fiber zone. At the distal end of CA3, mossy fibers form a distinct end-bulb.

Cell numbers of the principal cell populations of the dentate gyrus, hippocampus, and subiculum were estimated in Giemsa-stained sections of methacrylate embedded left hemispheres of four females and four males. Borders between cell populations (illustrated in **Figure 2**) are described in the results section on cytoarchitecture.

To obtain the estimates, we used the optical fractionator method (West et al., 1991) with StereoInvestigator 10 software (MBF Bioscience, Williston, VT, USA). Every second section of the mounted series, i.e., every 12th section, was sampled with 10 μm high disectors and 2 μm top guard zones at 210 μm intervals along the *x*- and *y*-axis. Section thickness was estimated at every 10th sampling site. Sampling parameters that differed between cell populations, the mean numbers of sampling sites, cells counted, total number estimates (based on number-weighted section thickness; Dorph-Petersen et al., 2001), and coefficients of error (CE) of the individual estimates for *m* = 0 (Gundersen et al., 1999; Slomianka and West, 2005) are listed in **Table 2**.

**Table 2:** Unilateral hippocampal principal cell numbers (rounded to the next 1000) in the eastern rock sengi hippocampus and sampling parameters (males, *n* = 4; females *n* = 4).

	Mean	SD	Mean CE (*m* = 0)	CE^2^/CV^2^	Counting frame size	Sections assessed Mean and range	Cells counted Mean and range
**Dentate gyrus granule cells**
All animals	3929000	857000	0.10	0.20	7 μm × 7 μm	9.6	235
Females	3618000	733000	0.11	0.30		8–11	155–234
Males	4240000	958000	0.08	0.13			
**Hilus/CA4 cells**
All animals	201000	39000	0.12	0.38	27 μm × 27 μm	8.1	178
Females	191000	52000	0.12	0.19		7–9	154–212
Males	212000	22000	0.12	1.31			
**CA3 pyramidal cells**
All animals	218000	44000	0.12	0.33	25 μm × 25 μm	8.3	165
Females	211000	43000	0.12	0.35		7–9	116–213
Males	224000	50000	0.11	0.25			
**CA1 pyramidal cells**
All animals	756000	124000	0.10	0.35	13 μm × 13 μm	10.4	153
Females	700000	149000	0.10	0.21		9–11	120–180
Males	813000	73000	0.10	1.12			
**Subicular cells**
All animals	137000	33000	0.10	0.18	28 μm × 28 μm	10.0	148
Females	150000	33000	0.10	0.23		9–11	125–224
Males	124000	33000	0.11	0.16			

Cell numbers of PCNA-positive (PCNA+) proliferating cells and doublecortin-positive (DCX+) type 2b-progenitors and young neurons were estimated in the immunohistochemically stained sagittal series of the right hemispheres. PCNA+ cells were counted exhaustively using area and thickness sampling fractions of 1, but omitting cells in the top focal plane of the sections from the counts. DCX+ cells were counted using a 30 μm × 30 μm unbiased counting frame applied at steps of 150 μm along both the *x*- and *y*-axis. The thickness sampling fraction was again 1, omitting cells in the top focal plane from the counts.

The ratio CE^2^/CV^2^ (CV: group standard deviation/group mean) was calculated to ascertain the contribution of the estimation procedure to group variances (**Tables 2 and 4**). To compare AHN of the sengi with other species, we normalized cell counts by dividing them by the number of resident granule cells (Amrein et al., 2011).

### AGE ESTIMATION

Eastern rock sengis are seasonal breeders and pups are born between September and March (Rathbun, 2005). Based on the time of capture (September), we expect that none of the animals in this sample is younger than 6 months or older than 12 months. We measured lens weight and bone lines (Cavegn et al., 2013), calculated the means for each measurements and assigned a rank as a percentage of the means. Ranks were linearly transformed into tentative ages in months.

### STATISTICS

The degrees of divergence/convergence of connections within the hippocampal region were calculated by dividing the number of cells in the projecting populations by the number of cells in the target populations for the eastern rock sengi and seven other species for which data sets obtained by design-based stereological techniques were available (see **Table 3**). The definitions of hippocampal fields in these studies were compatible with those used here. In particular, if a CA4 (a reflected blade of the CA3 pyramidal cell layer, Rosene and van Hoesen, 1987) was present, it was included in the cell counts of the hilus. A one-way ANOVA (SPSS 19, IBM SPSS Statistics) was used to test degrees of divergence/convergence for main species effects, with Bonferroni-corrected *post hoc* testing for differences between species pairs. *P*-values less than 0.05 (two-tailed) were considered significant.

**Table 3:** The degree of convergence or divergence (source cell number/target cell number) along the chain of hippocampal projections from the dentate granule cells to the subiculum. Bold numbers are statistically different from the value observed in the eastern rock sengi (*P*-values in parenthesis).

	DG → hilus/CA4	DG → CA3	CA3 → CA1	CA1 → subiculum
**Eastern rock sengi** (*Elephantulus myurus*)	19.6	18.4	0.29	5.8
**House mouse** (*Mus musculus*)^1^	24.6 (0.985)	**3.84** (<0.001)	**0.73** (<0.001)	**1.4** (<0.001)
**Brown rat** (*Rattus norvegicus*)^2^	20.9 (1.000)	**4.8** (<0.001)	**0.78** (<0.001)	**1.1** (<0.001)
**Harvest mouse** (*Micromys minutus*)^3^	**45.5** (<0.001)	**4.4** (<0.001)	**1.1** (<0.001)	**1.4** (<0.001)
**Dog** (*Canis lupus familiaris*)^4^	24.4 (0.788)	**6.1** (<0.001)	0.33 (1.000)	**1.4** (<0.001)
**Domestic pig** (*Sus scrofa*)^5^	**6.0** (<0.001)	**6.9** (<0.001)	0.37 (1.000)	**1.9** (<0.001)
**Tree shrew** (*Tupaia glis*)^6^			**0.73** (<0.001)	**2.1** (<0.001)
**Rhesus monkey** (*Macaca mulatta*)^7^	20.7 (1.000)	16.4 (1.000)	0.38 (1.000)	**2.4** (<0.001)
**Human** (*Homo sapiens*)^8^	**11.4** (<0.001)	**6.56** (<0.001)	0.24 (1.000)	**2.6** (<0.001)

1 *n* = 7, Sources: Fabricius etal., 2008

2 *n* = 15, Sources: West etal., 1991; Hosseini-Sharifabad and Nyengaard, 2007; Fitting etal., 2010

3 *n* = 5, M.J. West and L. Slomianka, unpublished data

4 *n* = 10, Sources: Siwak-Tapp etal., 2008

5 *n* = 5, Sources: original data kindly provided by the authors, Holm and West, 1994

6 *n* = 11, Sources: Keuker etal., 2004

7 *n* = 8, Sources: Keuker etal., 2003a

8 *n* = 73, Sources: West and Gundersen, 1990; West, 1993; Simic etal., 1997; Harding etal., 1998; Korbo etal., 2003

R (version 2.15.3) was used for the following analysis. The relationship between species and hippocampal cell population sizes was visualized with correspondence analysis (MADE4 R package, Culhane et al., 2005), which is similar to principal components analysis, but uses a weighted Euclidean distance to account for large differences in the absolute size of the neuron populations. Values for each animal were scaled by subtracting the mean of all principal neuron populations of that animal and dividing by their standard deviation. All animals therefore have cell counts with a mean of zero and a standard deviation of one across regions, but the relative differences between regions for each animal are retained. Only animals that had values for all cell populations that were estimated in the sengi were included.

Correlations between tentative age, and the numbers of PCNA+ proliferating cell, DCX+ young neurons and apoptotic cells were tested using Pearson’s r (SPSS 19, IBM SPSS Statistics). In that a negative correlation with age and positive correlations between the three cell populations were expected, one-tailed tests were performed and *p*-values less than 0.05 were considered significant.

Gender differences were tested using a one-way ANOVA (SPSS 19, IBM SPSS Statistics) with or without tentative age as a covariate. *P*-values less than 0.05 (two-tailed) were considered significant.

## RESULTS

All descriptions refer to the typical pattern observed at mid-septotemporal levels of the dentate gyrus, hippocampus, and subiculum. Septotemporal changes or gradients are mentioned after the description of the typical pattern.

### HIPPOCAMPAL CYTOARCHITECTURE

#### Dentate gyrus

**FIGURE 3:**
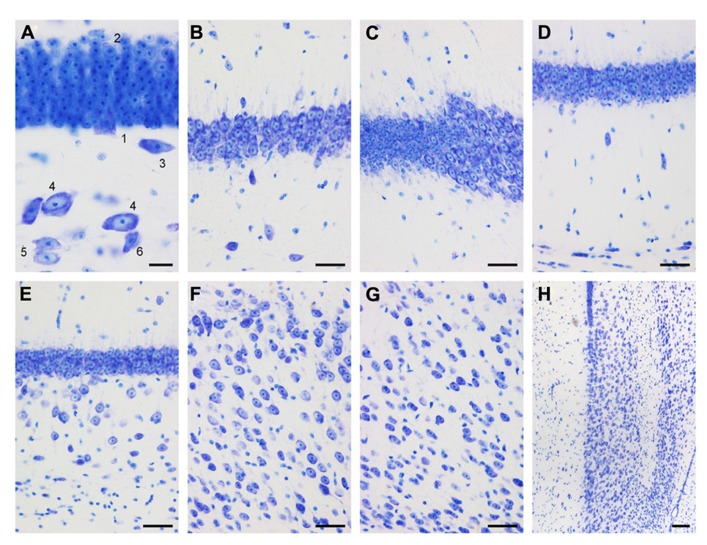
**Mid-septotemporal (unless noted otherwise) dentate gyrus and hippocampus in plastic embedded 20 μm-thick horizontal sections.** **(A)** Composite of two focal planes of the dentate gyrus granule cell layer, hilar plexiform layer (*hpl*), and hilar polymorphic cell layer (*hpcl*). Cell 3 was cloned into this image from an adjacent field of view. 1, pyramidal (basket) cell; 2, large granule cell; 3, spindle-shaped cell of the *hpl*; 4; large polygonal neurons; 5, ovoid neurons, the dominant population of the *hpcl*; 6, small dark triangular cell. **(B)** CA3. **(C)** Transition from CA3 to CA1. **(D)** Composite of two focal planes of the CA1 pyramidal cell layer (*CA1pcl*), stratum oriens (*CA1so*), and stratum oriens/alveus border (*so*/a). **(E)** Temporal *CA1pcl*. **(F)** Proximal subicular cell layer (*Scl*). **(G)** Distal *Scl*. **(H)** Temporal subiculum. Scale bars: **(A)** 20 μm; **(B–G)** 50 μm; **(H)**, 100 μm.

Small, round cells with a preference to be located either superficially or at depth are the most common type of neuron in the cell-sparse dentate molecular layer (*ml*). The granule cell layer (*gcl*) is 12–15 cells wide and has sharp boundaries to both the molecular cell layer and, in particular, the hilus (**Figure 3A**). The layer only forms distinct crests at the septal pole of the dentate gyrus (**Figure 2B**). Granule cells are densely packed and tend to form columns that span the *gcl*. Their large nuclei with one distinct and large nucleolus – a characteristic of all neuronal cell populations in the sengi hippocampus – are surrounded by a very narrow rim of cytoplasm. Pyramidal-shaped cells are embedded in the lower margin of the *gcl* (1, **Figure 3A**), while ovoid cells slightly larger than granule cells and with a larger cytoplasm are found at the upper *gcl* border (2, **Figure 3A**). A narrow hilar plexiform layer (*hpl*), which contains rare spindle-shaped cells that are oriented parallel to the *gcl* (3, **Figure 3A**) and rare ectopic granule cells, delimits the *gcl* from the hilar polymorphic cell layer (*hpcl*). Immediately below the *hpl*, a sparse population of large polygonal cells (4, **Figure 3A**), with 2 or 3 large primary dendrites in the plane of the section, gradually merges with the dominant cell population of the loosely packed *hpcl* – slightly smaller ovoid to polygonal cells with 3 or more primary dendrites extending from their soma (5, **Figure 3A**). A third population is formed by distinctly smaller and darker staining cells of typically triangular appearance (6, **Figure 3A**) that are scattered throughout the *hpcl*.

The CA3 pyramidal cell layer (*CA3pcl*) inserts into the *hpcl* close to the suprapyramidal limit of the *hpcl* (**Figures 2B–E**). While the *hpcl* forms a continuous wide band septally (**Figures 2B,C**), it is separated into two tiers temporally (**Figures 2D,E**), leading us to believe that the dominant cell population in the *hpcl* represents modified pyramids of a reflected blade of the *CA3pcl*. This interpretation is supported by the distribution of mossy fiber terminals (**Figure 2F**) that form a dense narrow band below the *gcl* thereby delimiting cells embedded in the band from the deeper part of the *hpcl*.

#### Hippocampus, CA3

The CA3 stratum lacunosum moleculare (*CA3slm*) is characterized by a distinctly higher density of glial cells than in the subjacent layers and frequent very small ovoid neurons. A few larger neurons align with the border between the *CA3slm* and CA3 stratum radiatum (*CA3sr*). The *CA3pcl* forms a typically four cells deep, dense band (**Figure 3B**) that widens considerably (**Figures 2B–D**) both at the transition to the *hpcl*, where the *CA3pcl* fans out into the *hpcl*, and to the CA1 pyramidal cell layer (*CA1pcl*). Few dark polymorphic cells are embedded in the deep part of the *CA3pcl*. Proximally, i.e., close to the dentate gyrus, ovoid, spindle-shaped, and small, dark pyramidal neurons are common (**Figure 3B**) in the adjacent CA3 stratum oriens (*CA3so*) and *CA3sr*. Their density decreases distally toward the transition to CA1. Distally, large round cells are seen in the *CA3so* and a group of smaller cell bodies of often spindle-shaped but variable morphologies is scattered close to the border between *CA3so* and the alveus. Their density decreases toward proximal CA3. Gradients in the distribution of cells outside the *CA3pcl* largely reflect differences in the densities of cells that express markers of inhibitory neurons (see below).

Cytoarchitectural characteristics of CA3 are conserved along the septotemporal axis of CA3. However, in proportion to CA1, CA3 shortens septally along its proximo-distal axis while it elongates temporally (**Figures 2B–E**).

#### Hippocampus, CA1

Similar to the *CA3slm*, the CA1 stratum lacunosum moleculare (*CA1slm*) is glia-rich but small neurons are rare. Spindle-shaped cells are found at the border between the *CA1slm* and the CA1 stratum radiatum (*CA1sr*) and align with it. The transition from the *CA3pcl* to *CA1pcl* is marked by a sudden decrease of the thickness of the *CA1pcl* that also contains markedly smaller pyramidal cell bodies (**Figure 3C**). CA1 and CA3 pyramidal cells intermingle for a very short distance. The *CA1pcl* is densely packed and typically four cell bodies deep (**Figure 3D**) – temporally it tapers from the proximal to the distal end (**Figure 2D**). Very rare (one or two per section) darkly stained polymorph or pyramidal-shaped cells are embedded in the deep part of the *CA1pcl*. Neurons in the *CA1sr* and CA1 stratum oriens (*CA1so*) are rare, typically ovoid or spindle-shaped and oriented perpendicular to the *CA1pcl* (**Figure 3D**). The distribution of neurons along the CA1so/alveus borders mirrors that of CA3 with cells being more frequent in proximal CA1 than in distal CA1. The *CA1pcl* ends abruptly at the subicular border (**Figures 2B–D**). Although the subicular cell layer (*Scl*) undercuts CA1 slightly, it does so less than in laboratory mice or rats, and the border is typically very sharp throughout the radial extent of the deep hippocampal layers.

Cytoarchitectural characteristics of CA1 are conserved along most of the septotemporal axis (**Figure 2**). Only in the temporal one-quarter are scattered larger pyramidal cells located beneath the compact superficial layer (**Figures 2E and 3E**) – first in the immediate vicinity of the subiculum and later beneath the distal one-third of the *CA1pcl*. CA1 and CA3 pyramidal cells do maintain their distinct difference in size along the entire septotemporal axis of the hippocampus.

#### Subiculum

Due to oblique borders with CA1 and the presubiculum, the *Scl* forms a lozenge-shaped field (**Figures 2B–D**). It is divided into distal and proximal areas of roughly equal size. Proximally, a condensation of small neurons is seen at the border between the subicular plexiform layer (*Spl*) and *Scl* (**Figure 3F**). Beneath this condensed part of the proximal *Scl*, a visually homogenous population of neurons with large ovoid somata and one large apical dendritic trunk spans most of the *Scl* (**Figure 3F**). Some smaller and darker cells are scattered within this population and become more frequent at the *Scl*/alveus border. In the deep part of the *Scl* they are accompanied by a number of dark pyramidal-shaped somata. The superficial condensation of cells is absent in the distal part of the *Scl*. Cells are generally smaller and darker and morphologically more heterogeneous than in the proximal *Scl* (**Figure 3G**). Toward the alveus, the distal *Scl* is delimited by a band of small elongated dark neurons that continues morphologically unchanged into the adjacent presubiculum.

Septally, the distal part of the *Scl* expands to occupy most of the *Scl* and the deep band of cells that delimits the *Scl* from the alveus is absent. In the temporal one-quarter of the subiculum, the proximal part of the *Scl* expands until it spans the entire proximo-distal extent of the subiculum (**Figure 3H**).

### HIPPOCAMPAL CELL NUMBERS

The total number of neurons estimated to be present in the dentate *gcl*, hilus/CA4, *CA3pcl*, *CA1pcl,* and *Scl* of male and female animals are listed in **Table 2**. CE^2^/CV^2^ were typically below 0.5, suggesting that only a minor part of the group variances originated from the estimation procedure.

Estimates tend to be slightly higher in males (+6 to +17%) than in females (except for the subiculum: -18%). Significant sex differences could, however, not be observed for individual populations or total hippocampal cell number (*p* = 0.22–0.71) with the group sizes used.

Degrees of convergence and divergence are listed in **Table 3** and compared to published data sets of other species that allowed calculating at least two values. The eastern rock sengi most closely resembles the macaque monkey until the subiculum is reached. The degree of convergence of CA1 pyramidal cells onto subicular neurons by far exceeds the values observed in all other species. Opposing but non-significant differences in CA1 and subicular cell numbers in male and female sengis did not result in a gender difference in convergence [*t*(6) = -1.71 *p* = 0.14].

**FIGURE 4:**
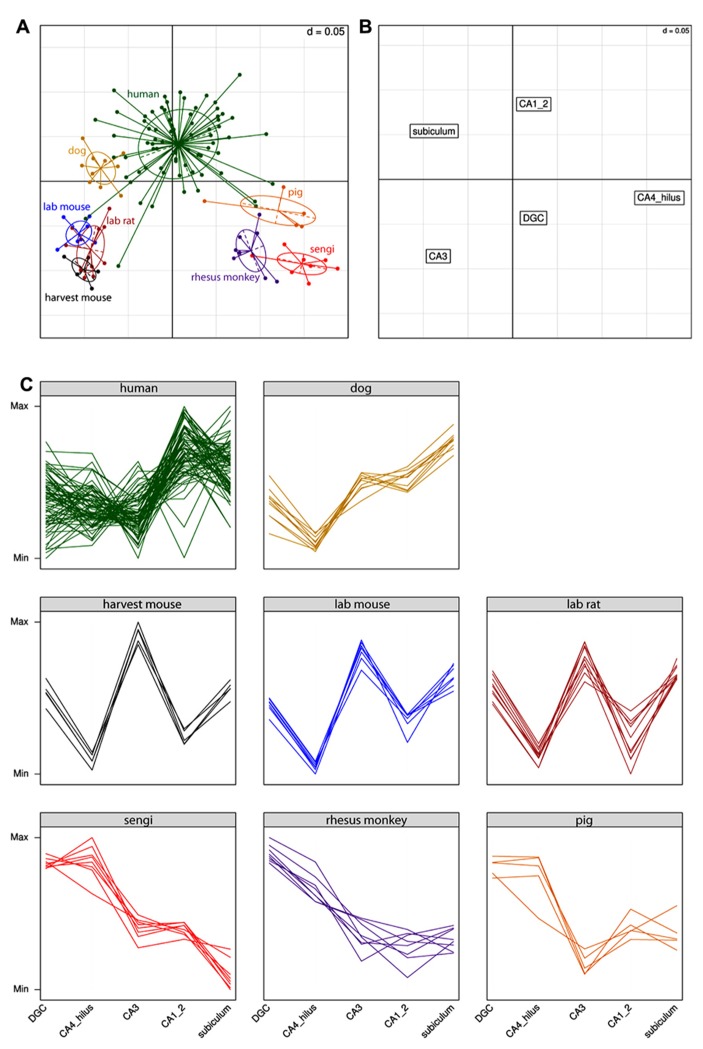
**Correspondence analysis (A,B) and species profile (C) plots show the relationships between cell counts and hippocampal fields.** Species form distinct clusters **(A)** with phylogenetically similar species such as the rodents clustering close together. The spatial arrangement of hippocampal fields in **(B)** can be used to determine which fields are driving the species clusters. For example, the rodents are located in the bottom left quadrant in **(A)**, as is the CA3 region in **(B)**. This means that rodents have relatively high numbers of cells in the CA3 (and relatively few cells in the CA4/hilus). These relationships can also be seen in the species profile plots **(C)**, where each line represents an individual animal. The *y*-axes range from the minimum to the maximum value for each hippocampal field, and therefore the most relevant comparisons are across species for a given field (e.g., it can be determined that rodents have relatively more cells in the CA3 than humans). Note that it is not possible to determine from these graphs whether mice have a greater number of cells in the CA3 or dentate gyrus (DGC), as each field uses a different scale.

Extending the analysis beyond the comparison of the sengi with other species, the results for the correspondence analysis are shown in **Figure 4**. Each point in **Figure 4A** is a single individual and the axes represent the reduction from five-dimensional space (where each hippocampal field is a separate axis) to two dimensions that capture most (91%) of the variation in the original data. This allows multidimensional patterns to be visualized in two dimensions with only a small amount of information loss. Individuals close to each other in **Figure 4A** have similar cell count profiles across regions, and it can be seen that the three rodent species cluster together, as do sengis and rhesus monkeys. The three remaining species (human, dog, and pig) are fairly distinct. **Figure 4B** plots the brain region profiles (a reduction from 126-dimensional space with one axis for each individual) and can be used to determine which brain regions are responsible for species clusterings, along with the eight panels of species profiles (**Figure 4C**). **Figures 4A,B** are on the same scale and are often plotted on one graph, but were separated for clarity. The rodents are spatially close to CA3 (they are in the bottom left quadrant in both graphs), and the interpretation is that rodents have a relatively high number of cells in the CA3. This is clear from the species profile plots where the rodents have a “spike” in the CA3. Similarly, the rodents are far away from the CA4/hilus (bottom right) and CA1/2 (top right) regions, meaning that they have relatively few cells in these regions, which can also be seen in the species profile plots. The interpretation is the same for the other species and regions (e.g., humans have relatively high cell counts in the CA1/2 while the sengi has relatively few cells in the subiculum).

### MARKERS OF NEURONAL FUNCTION

#### Calretinin

**FIGURE 5:**
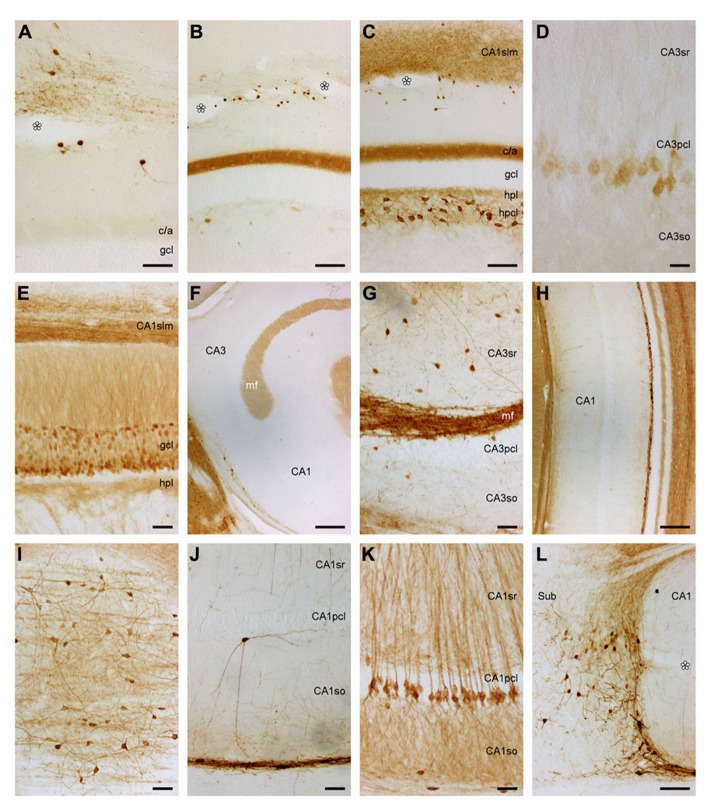
**Calretinin and Calbindin in the eastern rock sengi dentate gyrus, hippocampus, and subiculum. (A–D) Calretinin**. Asterisks: vessels in the obliterated hippocampal fissure. Scale bars: **(A)** 50 μm; **(B,C)** 100 μm; **(D)** 25 μm. **(A)** Septal dentate gyrus and border between CA1 stratum lacunosum moleculare (*slm*) and subicular plexiform layer. **(B)** Mid-septotemporal dentate gyrus and *CA1slm*. **(C)** Temporal dentate gyrus and *CA1slm*. **(D)** Calretinin+ cells in the deep pyramidal cell layer of temporal CA3. **(E–L)** **Calbindin**. Scale bars: **(E,G,I–L)** 50 μm; **(F,H)** 250 μm. **(E)** Heterogeneous distribution of calbindin in dentate granule cells. **(F)** calbindin+ cells are near absent in septal CA3. **(G)** Temporal CA3 **(H)** dense band of calbindin+ cells and processes at the stratum oriens/alveus border, but immunoreactive cells are otherwise near absent in CA1. **(I)** CA1, tangential section of stratum oriens/alveus border **(J)** Rare calbindin+ neuron deep to the CA1 pyramidal cell layer and fine plexus of fibers in the deep stratum oriens. **(K)** Calbindin+ deep pyramidal cells in temporal CA1. **(L)** CA1/subiculum border.

***Dentate gyrus***. The medial (*mpp*) and lateral (*lpp*) perforant pathways of the dentate *ml* are virtually unstained (**Figures 5A–C**), which agrees with the absence of stained cell bodies in the layer II of the medial and lateral entorhinal cortices. The *lpp* and *mpp* are sharply delimited from a well-stained commissural/associational zone (*c/a*, **Figures 5B,C**). Small strongly stained cell bodies are seen close to the hippocampal fissure (**Figures 5A–C**) and more frequently so over the suprapyramidal blade than the infrapyramidal blade of the *gcl*. One or two slender processes emanate from the cell bodies. When processes can be followed for some distance, they take on a weakly beaded appearance. Granule cells appear unstained. Rare (typically <1 per section) weakly stained pyramidal-shaped cell bodies are embedded in the lower aspect of the *gcl*. The *hpl* is weakly stained and merges without clear border with the *hpcl* that harbors few weakly stained, large, multipolar cell bodies (**Figure 5B**).

There are no staining gradients along the transverse axis of the dentate gyrus. Along the septotemporal axis, there are strong gradients in the appearance of the *c/a* zone and the *hpcl*. The *c/a* zone is very weakly stained septally (**Figure 5A**). Staining increases toward temporal levels (**Figures 5B,C**) and becomes moderate before stained cell bodies become apparent in the *hpcl* along the middle one-third of the dentate gyrus. Staining intensity of *hpcl* cell bodies increases markedly toward the temporal pole (**Figure 5C**).

***Hippocampus and subiculum***. With few exceptions, the hippocampus appears unstained throughout its transverse and septotemporal extent. Small intensely stained cells similar to those in the dentate *ml* are present in *CA3slm*. Weakly stained pyramidal cell bodies form a deep band in the temporal *CA3pcl* (**Figure 5D**). Darkly stained polymorphic cell bodies located close to the *CA1pcl* and *CA3pcl* are rare (typically <1 per section and <10 per series). A small field of fine terminal-like staining (**Figure 5A**) is seen at the transition from the *CA1slm* to the *Spl*. At the border between CA1 and the subiculum, a few small cells are embedded in this field (**Figure 5A**). The terminal-like staining expands temporally (**Figure 5C**) to fill the entire *CA1slm* and *Spl* at the temporal pole.

#### Calbindin

***Dentate gyrus***. The *ml* stains evenly light to moderate. Throughout most of the septotemporal extent of the dentate gyrus, the *gcl* has a trilaminar appearance: moderate to strongly stained superficial and deep cells are separated by a tier of very light or unstained cells. While the deep cells form a near continuous band, lighter cells scatter between the darkly stained cells of the superficial tier (**Figure 5E**). A few darker cells are also found in the light middle tier. The *hpl* appears unstained. The superficial *hpcl* appears similar to the *ml* and extends stained strands into the deep *hpcl* (**Figure 5E**) that coalesce to form the mossy fiber layer in CA3 (**Figure 5F**).

***Hippocampus and subiculum***. CA3 appears, with the exception of the mossy fiber layer, largely unstained (**Figures 5F,G**). Rare moderately stained cells are found at the *CA3so*/alveus border, which resemble those seen in CA1 (see below). Very rare (<1 per section) darkly stained cells are located around the *CA3sr*/*slm* border. Coinciding with the end of the mossy fiber layer, the number of cells located at the *so*/alveus border increases in CA1 (**Figures 5F,J**). They often appear bipolar in sections cut perpendicular to the hippocampal long-axis but multipolar in sections cut parallel to the alveus (**Figure 5I**) and extend a network of processes that is confined to the narrow band defined by the cell bodies (**Figures 5H,J**). At the CA1/subiculum border, cell bodies ascend toward the *CA1pcl* (**Figure 5L**). Fine processes and a few cell bodies extend into the proximal extreme of the subiculum (**Figure 5L**). Generally, stained cells are very rare in the remaining layers of CA1 (**Figure 5H**). A fine plexus of fibers is seen in the deep part of *CA1so* (**Figure 5J**). Rare strongly stained pyramidal or multipolar cells are seen in the *CA1pcl* (**Figure 5J**). Small moderately stained cells are seen rarely in the *CA1sr* and *CA1slm*. Well-stained fibers and fine particles stain the *CA1slm*.

Temporally, an increasing number of moderately stained cells is evenly distributed in the *CA3sr* and *CA3slm* (**Figure 5G**) and at the *CA1sr*/*slm* border. The temporal one-third of the *CA1pcl* is characterized by the gradual appearance of moderate to strongly stained deep pyramidal cells. Initially, they scatter as individual cells along the deep border of the *CA1pcl*, but distally and further temporally, they form a continuous deep band of the *CA1pcl* (**Figure 5K**).

#### Parvalbumin

**FIGURE 6:**
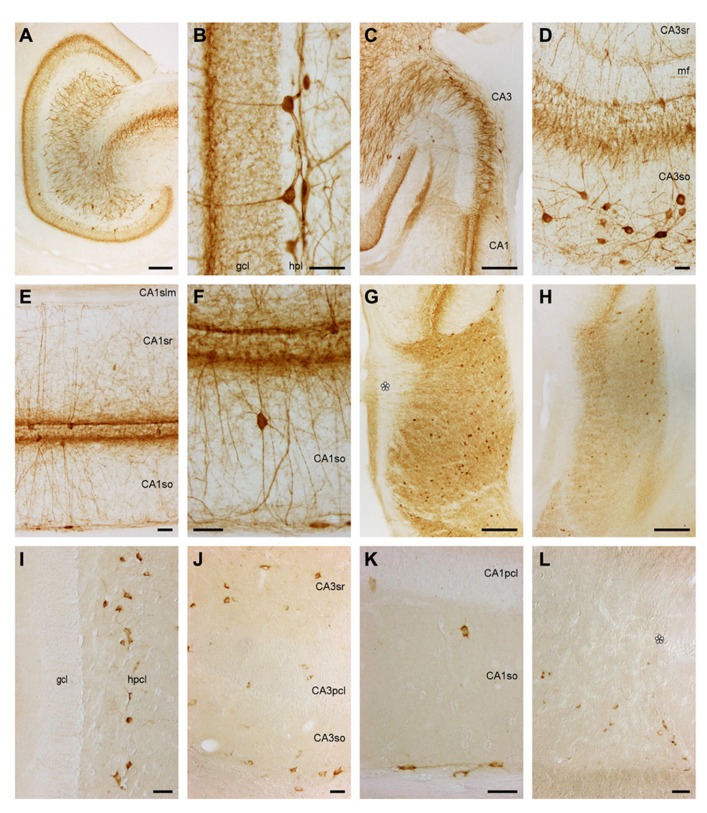
**Parvalbumin and Somatostatin in the eastern rock sengi dentate gyrus, hippocampus and subiculum. (A–H) Parvalbumin.** Scale bars: **(A,C,G,H)** 250 μm; **(B,D–F)** 50 μm **(A)** Mid-septotemporal dentate gyrus. **(B)** Septal granule cell and hilar plexiform layers. **(C)** Septal CA3. **(D)** Mid-septotemporal CA3 containing unusually many parvalbumin+ cells that reflect their morphological heterogeneity. **(E,F)** Mid-septotemporal CA1. **(G)** Septal subiculum. Beaded dendrites reach the pial surface in the proximal subiculum only (asterisk). **(H)** Temporal subiculum. **(I–L)** **Somatostatin**. Scale bars: **(I,J)** 50 μm **(I)** Dentate gyrus. Immunoreactive cells are only found in the hilar polymorphic cell layer. **(J)** CA3. **(K)** CA1. **(L)** Proximal subiculum (asterisk marks the end of the CA1 pyramidal cell layer).

***Dentate gyrus***. Stained cells or terminal-like staining are not seen in the *ml* (**Figure 6A**). Only at the border between the *ml* and *gcl*, a dense band of fine granules extends for a short distance into the layers forming the border (**Figures 6A,B**). Lower density, fine granules scatter also over the remainder of the *gcl*. Pyramidal-shaped somata are found between the deepest granule cells with a preference for the suprapyramidal blade of the *gcl* (**Figures 6A,B**) and extending beaded dendrites into the *ml*. The deep part of the *hpcl* harbors a dense network of coarse and fine beaded processes, a few of which reach into the otherwise unstained *hpl* and superficial *hpcl*. Parvalbumin+ multipolar cell bodies in the *hpcl* are located mainly beneath the suprapyramidal *gcl*.

Septally, pyramidal-shaped cells (**Figure 6B**) are more common and more evenly distributed. Horizontal fibers run through the *hpl* and superficial *hplc*. Horizontal cells are also present. Cell bodies in the *hpcl* become rare septally. In the temporal quarter, stained structures become rare until only even fine-grained staining remains over the *gcl* at the temporal pole.

***Hippocampus and subiculum***. In CA3, most cells are either located in *CA3so* or associated with the *CA3pcl* (**Figures 6C,D**). In *CA3so*, their dendrites form an irregular network, while most apical dendrites ascend straight through *CA3sr* (**Figure 6D**). They rarely enter *CA3slm*. Coarse and dense terminal-like staining is seen in *CA3slm* and at the deep and superficial borders of *CA3pcl* (**Figure 6D**). In CA1, cells are less frequent than in CA3. The vast majority is either associated with the *CA1pcl* or found at the *CA1so*/alveus border. They extend beaded dendrites into the adjacent layers (**Figures 6E,F**), but dendrites rarely cross into the *CA1slm* (**Figure 6E**). Parvalbumin+ cell morphologies at the *CA1so*/alveus border correspond to those of calbindin+ cells. Pyramidal-shaped cells are most common in association with the *CA1pcl*, but multipolar and horizontal cells are also present. Rare cells of *CA1so* and *CA1sr* are often bipolar and oriented perpendicular to the *CA1pcl* (**Figure 6F**). A light and fine punctuate stain fills the *CA1slm*. Denser and coarser particles together with fine fibers are seen throughout *CA1sr* and *so*, but concentrate above and below the *CA1pcl* (**Figures 6E,F**). Subicular parvalbumin+ cells are often pyramidal in the proximal *Scl*, but ovoid or multipolar in the distal subiculum. In both parts, they are located mainly in the deep *Scl* (**Figure 6G**). Dendrites extend to the pial surface proximally only, while immunoreactive cells are more frequent distally (**Figure 6G**). Coarse terminal-like staining fills the deep *Spl* and entire *Scl* (**Figures 6G,H**).

No gross changes in the staining pattern are seen along the septotemporal axis of CA3. Septally, cells associated with the *CA1pcl* become fewer, while terminal- and fiber-like staining is unchanged. Temporally, cells become rare in the distal subiculum (**Figure 6H**).

#### Somatostatin

***Dentate gyrus***. Immunoreactive cells are absent from the *ml*, *gcl*, and *hpl*. 30–50 large, bi- or multipolar cells scatter evenly throughout the superficial *hpcl* (**Figure 6I**). There is a clear septotemporal low to high gradient in cell density. Temporally, cells appear smaller and scatter evenly throughout the *hpcl*. Septally, cell morphology remains unchanged but staining intensity decreases.

***Hippocampus und subiculum***. Immunoreactive cells are scattered evenly along the *CA3so*/alveus border (**Figure 6J**). From there, they also extend in between CA3 and its reflected blade forming part of the *hpcl*. They stain weaker than in the dentate gyrus and their morphology is therefore difficult to determine. Both bipolar cells and cell with a prominent apical dendrite are among them. 5–10 per section are found scattered throughout the remaining layers of CA3, with a slight preference for the *CA3sr* (**Figure 6J**). In CA1, immunoreactive cell are almost exclusively found at the *CA1so*/alveus border (**Figure 6K**). Cell density is highest in the proximal part of CA1, and many cells have a bipolar appearance. Rare cells outside this zone (1 or 2 per section) are often located close to the deep *CA1pcl* (**Figure 6K**). The deep band of cells follows the CA1/subiculum border to the junction between *CA1pcl* and *Scl*. From there, they scatter in the deep one-half of the *Scl* without a proximal–distal gradient (**Figure 6L**).

Cell density increases from the septal to the temporal CA3, while it appears stable in CA1. In CA3, this increase is most pronounced in *CA3so* and *sr*, and it is accompanied by a dispersal of the cell band at the *so*/alveus border.

#### Neurogenesis

**FIGURE 7:**
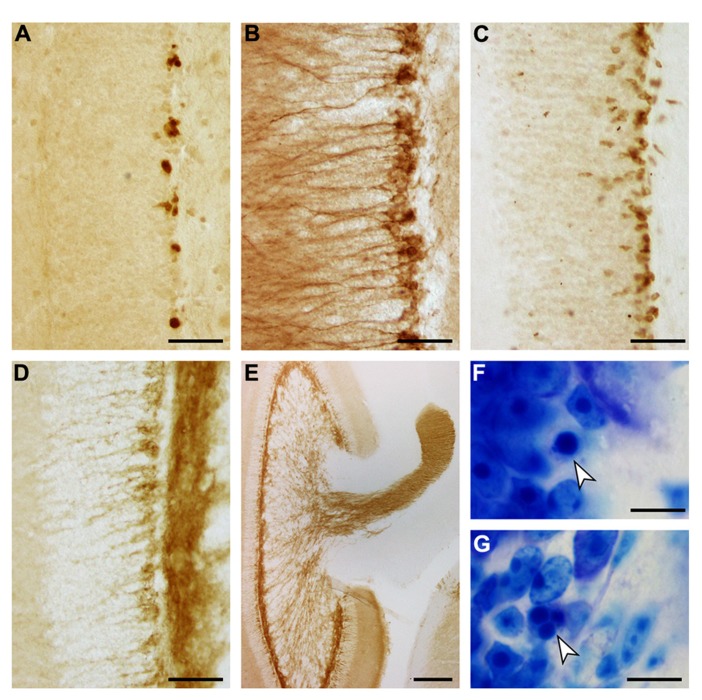
**Adult hippocampal neurogenesis in the eastern rock sengi dentate gyrus.** Images were taken in the septal hippocampus corresponding to **Figure 2B**. **(A)** PCNA+ cells. **(B)** DCX+ cells. **(C)** NeuroD+ cells. **(D)** PSA-NCAM+ cells and neuropil. **(E)** PSA-NCAM+ cells and mossy fiber terminal fields. **(F,G)** Pyknotic cells (arrows). Scale bars: **(A–D)** 50 μm; **(E)** 250 μm; **(F,G)** 10 μm.

The appearances of PCNA+ proliferating cell, DCX+, PSA-NCAM+ and NeuroD+ young neurons, and apoptotic cells are illustrated in **Figure 7**. Both proliferating cells (**Figure 7A**) and young neurons (**Figures 7B–D**) are distributed rather evenly along the lower margin of the *gcl*. Young neurons are rarely positioned further superficially than in the deep 2–3 cell tiers of the *gcl*. A decline in their numbers along the septotemporal axis of the *gcl* is noticeable but not pronounced. DCX did define cell bodies and dendrites more clearly than PSA-NCAM (**Figures 7B,D**), while PSA-NCAM defined the axonal fields of the cells in the hilus and CA3 better than DCX (**Figures 7D,E**). Based on the distribution of PSA-NCAM, the axons of young neurons are evenly distributed within the terminal fields of the mossy fibers (compare **Figures 2F and 7E**).

Number estimates corresponding to those presented for the principal cell populations are listed in **Table 4**. We did not observe any gender differences between the numbers of PCNA+ cells, DCX+ young neurons and apoptotic cells with [PCNA: *F*(1,9) = 1.22, *p* = 0.30; DCX: *F*(1,9) = 0.24; *p* = 0.64; Apo: *F*(1,9) = 1.21, *p* = 0.30] or without [PCNA: *F*(1, 10) = 0.92, *p* = 0.36; DCX: *F*(1, 10) = 0.25, *p* = 0.63; Apo *F*(1,10) = 0.24; *p* = 0.64] tentative age as a covariate. CE^2^/CV^2^ ratios were well below 0.5, suggesting that only a very small part of the group variances originated from the estimation procedure.

**Table 4:** Unilateral dentate gyrus neurogenesis related cell numbers in the eastern rock sengi (rounded to the next 100 for PCNA and doublecortin and the next 10 for apoptotic cells) and sampling parameters (CEs and CE^2^/CV^2^ rounded to two decimals).

	Mean	SD	Mean CE (*m* = 0)	CE^2^/CV^2^	Sections assessed Mean and range	Cells counted Mean and range
**PCNA+ proliferating cells**
All animals	12100	3600	0.04	0.02	13.3	1145
Females	10700	1100	0.04	0.13	11–15	905–1914
Males	13700	5000	0.04	0.01		
**Doublecortin+ cell of neuronal lineage**
all animals	41600	9600	0.09	0.15	13.2	158
Females	41900	6900	0.09	0.30	11–16	105–215
Males	41200	13100	0.09	0.08		
**Apoptotic cells**
All animals	800	210	0.05	0.04	12.8	78
Females	760	140	0.04	0.07	11–15	52–124
Males	850	280	0.05	0.03		

*All animals: *n* = 12, females: *n* = 6, males: *n* = 6.
*

The number of PCNA+ proliferating cell was highly correlated with both the numbers of DCX+ young neurons (*r* = 0.64, *p* = 0.007) and apoptotic cells (*r* = 0.72, *p* = 0.004). There was no correlation between the number of DCX+ young neurons and apoptotic cells (*r* = 0.35, *p* = 0.13). No correlation was found between tentative age and the numbers of PCNA+ proliferating cell (*r* = 0.055, *p* = 0.43) and apoptotic cells (*r* = -0.15, *p* = 0.32) while a trend toward a negative correlation was seen for DCX+ young neurons (*r* = -0.395 *p* = 0.10).

**FIGURE 8:**
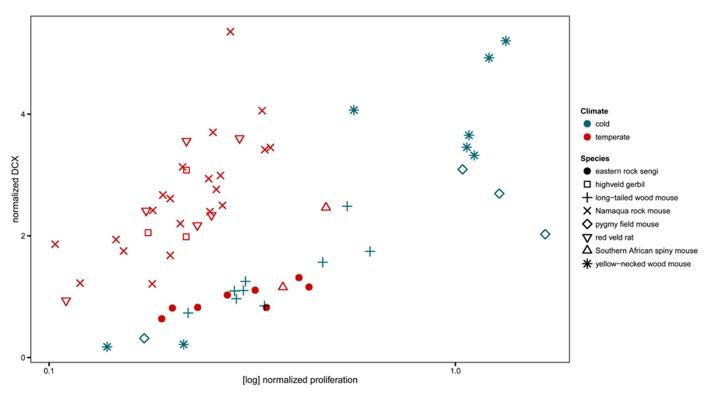
**Neurogenesis in the eastern rock sengi in comparison to southern African rodents from the same temperate climate, compared with neurogenesis in four murids from a northern cold climate (adapted from Cavegn et al., 2013.)** Neurogenesis data of sengis and spiny mice, both precocial species with exceptionally high numbers of granule cells, cluster with the species from the cold climate. For species comparisons, the numbers of proliferating cells (PCNA+ or Ki67+) and young cells of the neuronal lineage (DCX+) were normalized to total granule cell number.

The normalized numbers of PCNA+ cells in sengis fall within the range defined by all four species of murine rodents that had been captured in the same location. However, the normalized numbers of DCX+ cells were lower than in three of the murine species (**Figure 8**).

## Discussion

The eastern rock sengi hippocampus does show individual characters that associate it with any one of the phylogenetic groups that it has been related with. Their combination is, however, unique and, as will be discussed below, forms a distinct data point in the matrix of hippocampal characters that represent species with different phylogenetic and ecological backgrounds. Results will be discussed in the order in which they have been described.

### HIPPOCAMPAL CYTOARCHITECTURE AND CELL NUMBERS

Cytoarchitecturally, the eastern rock sengi hippocampus may, grossly speaking, be described as having a primate like dentate gyrus and CA3, to which a CA1 and subiculum that resemble those found in the rabbit or guinea pig (Geneser-Jensen et al., 1974; Geneser, 1987a) have been added. Cell numbers also reflect the qualitative similarity of the dentate gyrus and CA3 to that of the rhesus monkey. A high degree of convergence from granule cells to CA3 pyramids is shared by the two species. Recalling early comments on the size of the sengi hippocampus, the total number of granule cells in the sengi (3.9 million unilateral, bodyweight ~50 g) is higher than in marmoset monkeys (3.6 million unilateral, bodyweight ~250–500 g; Kozorovitskiy et al., 2005) and more than half of that in adult rhesus monkeys (7.7 million unilateral, bodyweight ~7 kg; Keuker et al., 2003a). The large sengi dentate gyrus is not accompanied by a large entorhinal cortex (Stephan, 1961).

Comparative aspects of the junction between the dentate gyrus and CA3 have been subject of early and sustained discussions (Lorente de Nó, 1934; Rosene and van Hoesen, 1987; Keuker et al., 2003b). It is generally accepted that primates show a reflected blade of the hippocampal pyramidal cell layer, a CA4 as defined by Rosene and van Hoesen (1987), that is not found in laboratory mice and rats, in which the deep hilus forms a polymorphic cell mass connectionally and cytoarchitecturally unrelated to CA3 pyramidal cells (Blackstad, 1956; Amaral, 1978). The definition of CA4 by Rosene and van Hoesen (1987) excludes the hilar polymorphic cells that Lorente de Nó (1934) for “didactic” rather than connectional or cytoarchitectural reasons included as a second reflected blade in his definition of CA4. A reflected blade of the hippocampal pyramidal cell layer is, with minor morphological variations on the theme found in primates, present in fox (Amrein and Slomianka, 2010), pig (Holm and West, 1994), guinea pig (Geneser-Jensen, 1972), and rabbit (Geneser, 1987b) – to name but a few species. Despite the close relation between CA4 and CA3, CA4 pyramidal cells have typically been grouped with hilar polymorphic cells in quantitative studies, presumably because a reliable border between CA3 and CA4 is often easier to define than the border between the *hpcl* and CA4. Unexpectedly, this apparently spurious assignment does result in very similar degrees of convergence from dentate granule cells to hilar/CA4 cells in species with and without a CA4 (laboratory mouse, laboratory rat, dog, rhesus monkey, and sengi). Only pigs and humans, both with a CA4, show a much smaller convergence. In contrast, more variation is seen in the convergence from the anatomically “cleanly” defined dentate granule cells to CA3 cells. The unexpected association of rats and mice with dog, rhesus monkey and sengi may be explained by septotemporal changes in dentate histoarchitecture. An increase in the cell number of the temporal hilus (Gaarskjaer, 1978) of rats and mice that cannot be accounted for by an increase in the number of mossy cells alone (Fujise and Kosaka, 1999) and concomitant changes in the conformation of the dentate-CA3 junction (Haug, 1974) suggest that a CA4-like cell population may be present temporally. Beyond the mossy fiber projection, back projections from proximal/ventral CA3 to the dentate gyrus (Li et al., 1994; Scharfman, 2007) may create a functional link (e.g., in dentate pattern separation, Myers and Scharfman, 2011) close enough to mediate a concerted phylogenetic development of CA4 cell numbers and dentate granule cell numbers.

Although the cytoarchitecture of the sengi CA1 is strikingly different from that of primates, dog and pig, the degree of divergence from CA3 to CA1 pyramids of all these species is similar and larger than in rat, mice and tree shrew. The correspondence analysis does identify CA1 as a region particularly large in humans, but it does not group humans with rhesus monkeys, which confirms earlier observations (Seress, 1988). Although the size of CA1 expands in primates (Stephan, 1983), this expansion does not set the rhesus monkeys apart from other taxonomic groups represented by pig and sengi.

The proximal and distal subicular divisions and their changes along the septotemporal axis in the sengi subiculum are similar to those in rats and mice, in which they correspond to subicular compartments with distinct connectional and physiological properties (O’Mara et al., 2001; Witter, 2006; Ohara et al., 2009). In sengis, subicular cell number is very small when compared to that of CA1, the main source of hippocampal subicular afferents. Extrahippocampal limbic system cell numbers are only available for two species (rats and humans) precluding an extended correspondence analysis of cell numbers. However, size indices based on volumes for the sengi retrohippocampal cortical (peri- and entorhinal cortex) and subcortical (septum) CA1 target areas are smaller than those for the hippocampus itself (Stephan, 1961), which would argue that they do not function as alternative, unusually large targets for CA1 projections. This is similar to the size relations between the entorhinal cortex and the dentate gyrus at the entrance to hippocampal circuitry and raises the question of the computational advantage of a large hippocampus when no commensurate changes are apparent in the sources and targets of hippocampal connections.

What is brought out by the comparative quantitative data is that generalized models of hippocampal function will have to be robust to up to fivefold differences in ratios between the numbers of interconnected cell populations. Furthermore, interspecific differences encompass opposing trends in the degrees of divergence and convergence both across the entire tri-synaptic loop as well as at specific steps along this path. Lastly, while similarities exist between sengi and taxonomically disparate groups in the quantitative composition of hippocampal principal neurons, sengis form a distinct cluster in the data space defined by quantitative relations between the major brain divisions that is clearly separated from, e.g., primates (de Winter and Oxnard, 2001; Oxnard, 2004). Similarly composed hippocampi have thus been embedded into quantitatively different higher level networks.

### MARKERS OF NEURONAL FUNCTION

#### Calretinin

Based on cellular morphology, location and staining gradients in the *c/a*-zone of the dentate *ml*, calretinin+ cells in the hilus are likely to represent mossy cells with projections that ascend beyond the location of the cell body, but with only sparse projections reaching the septal pole of the dentate gyrus. Cytoarchitecturally, the cell type that is calretinin+ in the temporal hilus is calretinin- in the septal hilus. A similar distribution of calretinin in mossy cells was seen in the mouse, hamster, gerbil, laboratory shrew, primates, and humans (Liu et al., 1996; Blasco-Ibáñez and Freund, 1997; Fujise et al., 1998; Murakawa and Kosaka, 1999; Jinno and Kosaka, 2006; Seress et al., 2008). Calretinin+ CA1 and CA3 interneurons that are present in the above mentioned species and in rat (Gulyás et al., 1992), monkey (Seress et al., 1993), and human (Nitsch and Ohm, 1995) are, with very rare exceptions, absent in the sengi. In contrast, small calretinin+ cells in the sengi dentate and CA3 molecular layers are rarely seen in adult individuals of other species. Their distribution in the adult sengi and gerbil (Murakawa and Kosaka, 1999) resembles that of calretinin+ Cajal–Retzius cells in the developing mouse hippocampus (Soriano et al., 1994; Berger and Alvarez, 1996; Jiang and Swann, 1997) and adult human hippocampus (Abraham and Meyer, 2003).

Calretinin+ CA3 hippocampal pyramidal cells have been reported during murine development (Jiang and Swann, 1997), but not in adult laboratory rats. A few calretinin+ proximal CA3 pyramidal cells were seen in laboratory mice (Fujise and Kosaka, 1999). They are present throughout CA3 in a subpopulation of pyramidal cells of the Guaira spiny rat (Fabene et al., 2001), and we also reported the presence of another calcium-binding protein, calbindin, in deep CA3 pyramidal cells in naked mole-rats (Slomianka et al., 2011). Lastly, calretinin is not present in cells of the deep *gcl* or in the *hpl* (including the subgranular zone, see discussion of neurogenesis).

#### Calbindin in principal cells

We recently noted a heterogeneous distribution of calbindin in the granule cells of three sympatric African murine rodent species (Cavegn et al., 2013) that in two of the species appears similar to that in the sengi, i.e., calbindin+ cells in superficial and deep cells of the *gcl* separated by a tier of very light or unstained cells. A morphological and functional heterogeneity defining a population at the superficial limit of the rat *gcl* has also been described (Williams et al., 2007; Larimer and Strowbridge, 2010). Notably, a preferential expression of calbindin in superficial granule cells is also seen in *FMR1* knockout mice (Real et al., 2011), a gene encoding FMRP (fragile mental retardation protein). A morphologically heterogeneous population of granule cells is present in the fox as well (Amrein and Slomianka, 2010). It has been suggested that connections between developmentally matched groups within the hippocampal principal cell populations form distinct information streams (Deguchi et al., 2011), and differential expressions of calcium-binding proteins add to the morphological and functional differences that may be reflections of such streams.

Superficial calbindin+ CA1 pyramidal cells are found in mouse, rat, fox, and primates (reviewed in Slomianka et al., 2011). This marker is not expressed by superficial pyramids in guinea pig and rabbit and is absent in the sengi too. In mice, the condensation of pyramidal cells into a compact layer and the expression of CA1 specific markers, including calbindin, are developmentally regulated by the expression of Zbtb20 (Nielsen et al., 2007, 2010; Xie et al., 2010; Rosenthal et al., 2012). In contrast, the absence of calbindin from superficial pyramidal cells in spite of a pronounced condensation of the cell layer suggests an independent development of these characteristics in the sengi, guinea pig and rabbit CA1. Mechanisms related to age- (Poitier et al., 1994; Villa et al., 1994; de Jong et al., 1996), activity- (Lowenstein et al., 1991; Maglóczky et al., 1997), or hormone-dependent (Krugers et al., 1995; Trejo et al., 2000) changes of calbindin content in hippocampal principal cells may mediate species-specific expression patterns. Deep calbindin+ pyramidal cells are present in CA1 of fox and primates and in the distal and temporal CA1 of mouse and rat. In these species, they form the deepest tier of pyramids in a temporally and distally wide and trilaminar *CA1pcl*. Deep calbindin+ pyramids in the sengi share the distal and temporal location, but they still form a compact band together with calbindin- superficial pyramidal cells.

#### Calbindin in interneurons

There is a very marked shift in the distribution of calbindin+ putative interneurons to the stratum oriens/alveus border in the sengi when compared to mouse (Jinno and Kosaka, 2006), rat (Sloviter, 1989; Tóth and Freund, 1992), dog (Hof et al., 1996), or primates (Seress et al., 1991; Sloviter et al., 1991). Cell morphologies in this zone correspond to those described in primates. The distinct plexus of fibers in the *CA1slm* suggest an origin from O-LM inhibitory neurons (Freund and Buzsáki, 1996; Wouterlood et al., 2001) which is supported by the appearance of the dendritic arbors of many calbindin+ cells at the *CA1so*/alveus border. Interneurons with selective axonal projections to the *CA1so* have not been described (Freund and Buzsáki, 1996). Although O-LM cell axons do arborize in the *CA1so*, this is a minor projection compared to the *CA1slm* in laboratory mice and rats. Cell morphologies and location of calbindin+ cells also correspond to septally projecting type II cells of Gulyás and Freund (1996). Outside the stratum oriens/alveus border, calbindin+ interneurons are rare or absent in most of the sengi dentate gyrus and hippocampus – a feature that the sengi shares with the Guaira spiny rat (Fabene et al., 2001). Only in temporal CA3 does their apparent number and distribution correspond to that in other species. In contrast to the dentate gyrus and hippocampus, proximal–distal and radial distributions as well as septotemporal changes of the distributions of calbindin+ cells in the subiculum corresponds well to those of mice (Fujise et al., 1995).

#### Parvalbumin

The laminar distribution of parvalbumin in sengi interneurons corresponds to that in the dentate gyrus and hippocampus of rat (Nomura et al., 1997), mouse (Jinno and Kosaka, 2006), dog (Hof et al., 1996), tree shrew (Keuker et al., 2003b), or monkey (Seress et al., 1991). They still appear, to us, less frequent than in these species, although the difference is much less pronounced that that for calbindin and calretinin and would require robust quantitation to draw firm conclusions. Much of the dense parvalbumin+ terminal staining reflects, like in other species, the distribution of axons of parvalbumin+ chandelier and pyramidal basket cells (Freund and Buzsáki, 1996; Sik et al., 1997). The distribution of parvalbumin in the subiculum follows, like that of calbindin, closely the pattern described in mice (Fujise et al., 1995). While parvalbumin appears more resilient to phylogenetic change than calretinin or calbindin in eutherian mammals, the distribution is restricted to fewer cell types in prototherian echidna (Hassiotis et al., 2005).

#### Somatostatin

The cellular distribution of somatostatin in the sengi hippocampus is similar to that of the rat and mouse (Köhler and Chan-Palay, 1982; Bakst et al., 1986; Buckmaster et al., 1994), guinea pig and rabbit (Buckmaster et al., 1994), pig (Holm et al., 1992), and human (Chan-Palay, 1987), although neurons are generally more common in the stratum oriens of these species than in the sengi. In the dentate gyrus, they are likely to correspond to cells with axonal arbors in the perforant path zones (HIPP cells, Freund and Buzsáki, 1996). We have not been able to show a plexus of somatostatin containing fibers that is present in the dentate *ml* or *CA1slm* of mouse and rabbit (Buckmaster et al., 1994). Somatostatin containing cells at the *so*/alveus border of CA3 and CA1 are likely to represent LM- and O-LM neurons, respectively (Freund and Buzsáki, 1996). They have also been found to co-localize with calbindin (Wouterlood et al., 2001).

### NEUROGENESIS

Home ranges of eastern rock sengi females are typically smaller than those of males (Ribble and Perrin, 2005). In that AHN did not differ between males and females, spatial requirements associated with the absolute size of the home range are unlikely to be related to AHN. We have recently shown (Cavegn et al., 2013) that Muridae trapped in the same habitat (Köppen–Geiger climate classification Cwa, Peel et al., 2007) as the sengis did differ in proliferation (lower) and neuronal differentiation (higher) from Muridae that were collected in a different, European habitat (Köppen–Geiger climate classification Dfb). This difference was consistent across multiple species that belonged to different taxonomic subunits and that also differed in, e.g., habitat use and social organization. The sengi does not group with most of the sympatric Muridae, but instead associates with European Muridae. Only southern African spiny mice showed AHN related cell counts close to those of the sengi. Spiny mice and sengi share the traits of exceptionally high numbers of granule cells (Cavegn et al., 2013) and precociality. Current data cannot address how much of the observed variation in AHN of sengis and spiny mice could be related to phylogeny, to anatomical context of AHN or to demands on hippocampal information processing mediated by the young neurons that relate to their ecology. Also, the regulation of markers that are expressed during specific stages of neuronal differentiation differs in the sengi from that seen in other species. Calretinin is expressed during the late post-mitotic differentiation of granule cells in mice (Brandt et al., 2003; Kempermann et al., 2004). Calretinin+ cells are not seen in numbers or morphologies that correspond to those of DCX+ or PSA-NCAM+ cells in the dentate subgranular zone and deep *gcl*. The expression of calretinin is apparently not part of neuronal differentiation in the sengi.

Similar size progressions of the hippocampus and dentate gyrus in sengis and primates do not relate to AHN in similar ways. AHN is not only low in primates, but also characterized by an extended period of differentiation of the newly formed cells of neuronal lineage (Ngwenya et al., 2006; Kohler et al., 2011). Both the numbers of proliferating and differentiating cells and their ratio in the sengi fall within the ranges seen in murine rodents. Clearly, a large hippocampus relative to body size does not necessarily obviate AHN.

## CONCLUSION

The distinct positions of species, including a clustering of mice and rats, in the correspondence analysis point toward the existence of distinct patterns of quantitative relations between hippocampal principal cell populations. If these patterns relate to the phylogenetic history of the species and/or to demands on hippocampal information processing related to species ecology remains an open question. de Winter and Oxnard (2001) showed that quantitative relations between even major brain divisions could group species by lifestyle despite different phylogenetic histories. This is likely to be more common as anatomical resolution and, thereby, functional specificity increases. In our data, this is brought out by the separation of the human and rhesus monkey hippocampus and the association of rhesus monkey, pig, and sengi despite their disparate phylogenetic histories. The scarcity of calbindin+ and calretinin+ interneurons in, in particular, CA1 of the sengi hippocampus represent an additional tier of specialization that separates the sengi from species which appear similar based in quantitative relations between principal cells.

In the context of sengi hippocampal specializations, “normal, mouse-like” quantitative data on sengi AHN are an almost surprising relief. However, the sengi affiliates with our sample of European mice instead of most of the sympatric mouse species, which again raises the question if differences relate to anatomical context and/or selective pressures on AHN related to sengi ecology.

The most intriguing question remaining is why the sengi hippocampus is so large when the cortical and subcortical areas that are hippocampal sources of afferents and targets of efferents are not. We pointed out that generalized models of hippocampal function need to be robust to large differences in convergence and divergence along the intrahippocampal pathways. In addition, such models may have to endow large hippocampi with intrinsic computational abilities which provide selective advantages that, based on our current knowledge, are independent of the number of available input and output channels.

## Conflict of Interest Statement

The authors declare that the research was conducted in the absence of any commercial or financial relationships that could be construed as a potential conflict of interest.
